# Multi-omics insights into beagle dog fed with a sucking-rewarded automatic feeding device

**DOI:** 10.3389/fped.2024.1467581

**Published:** 2024-11-28

**Authors:** Yang Jiao, Xin Wang, Aizhen Yu, Li Wu, Hongping Li

**Affiliations:** ^1^ Kashgar People’s Hospital, Kashgar Prefecture, Xinjiang Uygur Autonomous Region, Kashgar, China; ^2^ Neonatology Department, Affiliated Shenzhen Children's Hospital of Shantou University Medical College, Shenzhen, Guangdong, China

**Keywords:** preterm infants, automatic feeding, multi-omics, sucking, beagle dogs

## Abstract

**Background:**

Facilitating the development of the sucking function in early stages of preterm infants holds substantial potential for influencing their long-term outcomes. To this end, our team has devised a sucking-rewarded automatic feeding device specifically tailored for preterm infants. The present study is designed to investigate the impacts of this innovative device, utilizing a multi-omics profiling approach, on beagle dogs as a surrogate model.

**Methods:**

This study involved seven-day-old male newborn beagle puppies, carefully selected and matched in terms of body weights. The participants were stratified into two groups: the experimental group (AFG, sucking-rewarded feeding group) and the control group (PFG). After a 14-day intervention period, fecal and blood samples were systematically collected from each dog. The collected samples were then subjected to distinct profiling analyses, encompassing the assessment of gut microbial composition, plasma metabolic profiles, and proteomic expression profiles.

**Results:**

The gut microbial data showed a significant difference between the AFG and PFG groups based on Bray-Curtis dissimilarity (*P* = 0.048), and the relative abundance of *Lactobacillus* was significantly more abundant in the AFG group compared to the PFG group. The significantly different metabolites between the two groups were enriched in functional metabolic pathways related to amino acids, fatty acid metabolism, and the nervous system. Notably, neurotransmitter L-glutamic acid was significantly up-regulated in the AFG group. Moreover, the significantly different proteins between the two groups were enriched in GO terms related to oxygen transport, oxygen binding, iron ion binding, hemoglobin complex, and heme binding. Among them, proteins A0A8C0MTD2, P60524, P60529 were significantly up-regulated in the AFG group. Notably, *Lactobacillus*, L-glutamic acid, A0A8C0MTD2, P60524, and P60529 were correlated with each other through correlation analysis, these molecules play important roles in the neural function and neurodevelopment.

**Conclusion:**

Our investigation elucidated discernible modifications in gut microbial composition, plasma metabolic profiles, and proteomic expression patterns in beagle dogs subjected to the sucking-rewarded automatic feeding device.

## Introduction

The ontogeny and refinement of sucking behavior in preterm infants significantly contribute to their overall developmental trajectory (1). Sucking behavior typically emerges around the 20th week of gestation, evolving from an initial immature pattern to a rhythmic one, and ultimately culminating in a sophisticated sucking-swallowing-breathing sequence conducive to oral feeding (1, 2). This intricate developmental process is intricately associated with the maturation of the neural network regulation function of the sucking central pattern generator (3). This inherent competence in sucking naturally advances with gestational age, with medical interventions offering the potential to expedite its progression at earlier developmental stage (4–6). The successful transition to oral feeding is contingent upon the maturation of the sucking-swallowing-breathing function, typically attaining completion around the 34th week of gestation (7–9). Preterm infants born before this critical gestational milestone face an elevated risk of aspiration during oral feeding, potentially exacerbating their medical condition. Current clinical practices frequently involve administering nutrition through a gastric tube, thereby bypassing the oral cavity and esophagus to directly infuse the stomach.

The protracted underutilization of sucking ability in preterm infants, particularly up to 34 weeks, may result in enduring feeding challenges extending into infancy (10, 11). Prolonged dependence on gastric tube feeding may heighten the susceptibility to respiratory diseases and impede brain development in these infants (4, 12, 13). The early introduction of sucking and oral stimulation, encompassing tactile and thermal elements, has demonstrated efficacy in fostering the development and maturation of sucking ability, potentially abbreviating the transition time from tube to complete oral feeding in preterm infants (14, 15). Sucking activities also contribute to the stimulation of gastrointestinal hormone secretion and the maturation of digestive function (16). Furthermore, sucking has been correlated with enhanced sleep quality, thereby contributing to improved gastrointestinal motility (17, 18). In current clinical protocols, the traditional feeding paradigm involves the direct infusion of nutrition into the stomach, typically through the administration of prescribed milk formula at fixed intervals by healthcare professionals, often overlooking infants' hunger cues. Conversely, the promotion of oral sucking function through responsiveness to hunger signals has proven effective (19), with sucking activity identified as a crucial hunger signal. Building upon this concept, our research team has innovatively designed a sucking-rewarded automatic feeding device for preterm infants. The device features a specialized nipple capable of detecting sucking activity when placed in the infant's mouths and triggers the delivery of a predetermined amount of milk into the stomach via a gastric tube. However, a comprehensive assessment of its impact on preterm infants is imperative. To address this, we propose the application of multi-omics profiling, a pioneering methodology aimed at thoroughly measuring, analyzing, and integrating various strata of biological molecules. This approach provides a unique opportunity to comprehensively understand and derive profound insights into the physiological status of humans (20, 21). In this study, we employed multi-omics profiling, encompassing proteome, metabolome, and microbiome analyses, to scrutinize the effects of the device on beagle dogs as part of our investigative process.

## Materials and methods

### Characteristics of the sucking-rewarded automatic feeding device

The sucking-rewarded automatic feeding device is composed of four main modules: a specialized nipple, a suction detector, a control unit, and a milk supply module. The dedicated nipple senses the stresses and deformations generated during sucking, which are transmitted to a pressure sensor to produce an electrical signal. Subsequently, the controller adjusts the speed of the electric motor in the supply device based on the strength of the received electrical signals, ensuring that the appropriate amount of milk is injected into the feeding tube placed in the stomach.

The sucking action collection utilizes a pressure monitoring mode, with the pacifier pressure collection pipeline leading to the machine-end pressure collection interface. It is then transformed into an analog electrical signal through a pressure sensor for display on an LCD screen. Signal conversion and calculation are carried out using a high-speed 32-bit single-chip microcomputer, with a communication speed of 10 k per s, significantly higher than the human eye's 50 Hz recognition ability. Consequently, the acquisition process swiftly translates the sucking action into waveform amplitude changes without delay.

### Animal experiment

Given the similarity in gastrointestinal peristalsis mechanisms between dogs and humans during digestion (22), beagle dogs were selected as the experimental subjects in this study to explore distinctions between feeding using an automatic feeding instrument and traditional syringe-based gavage feeding. Seven-day-old male newborn beagle puppies, matched in body weights, were chosen as the study participants. They were stratified into two groups: the experimental group (AFG, active feeding group with the sucking-rewarded feeding device) comprising 5 puppies and the control group (PFG, passive feeding group) consisting of 5 puppies. The experimental group received nourishment through the automatic feeding device, while the control group underwent gastric tube feeding administered by syringe. Both groups were provided with formulated milk powder, prepared according to the product instructions. The feeding volume for each puppy was set at 40 ml per feeding session, comprising 10 ml of iohexol and 30 ml of prepared milk. All procedures involving animal studies adhered to ethical standards and were conducted in accordance with the guidelines outlined by the animal ethical committee (Shenzhen TopBiotech Co., Ltd., China, No. TOP-IACUC-2022-0161).

Following each feeding session, the puppies were gently settled into a custom-made, warm, and dry cardboard box. A soft cushion was positioned in front of their bodies, creating a comfortable and unrestrictive for the puppies to sit quietly without undue restraint. Within the box, the puppies were positioned at an inclination of approximately 30–50 °C, with their heads elevated and hips lowered. To facilitate acclimatization to this setup, the puppies underwent a training period of 5 days prior to the actual study. Throughout this adaptation phase, familiar caretakers accompanied the puppies to prevent any distress and ensure a sense of security. This approach aimed to create a conducive post-feeding environment that prioritized well-being and comfort of the study subjects.

### Sample collection

Following a 14-day intervention period, fecal samples were meticulously collected from each dog using sterile gloves and collection tools. Approximately 200 grams of fecal material were aseptically transferred to sterile, pre-labeled containers and promptly stored at −80 °C to preserve the integrity of the DNA until extraction.

Concurrently, blood samples were obtained from each dog. Whole blood was drawn into EP tubes. After blood collection, a controlled centrifugation process was employed to separate plasma. The resulting plasma samples were carefully stored at −80 °C to maintain their quality until further analysis. The meticulous sample collection and preservation process aimed to ensure the reliability and integrity of biological materials for subsequent investigations.

### Gut microbiome sequencing and analysis

Microbial DNA extraction was conducted using the QIAamp DNA Mini kit, and the concentration and purity of the extracted DNA were assessed using the NanoDrop One (Thermo Fisher Scientific, MA, USA). The V3-4 variable region of the 16S rRNA gene was targeted for amplification using the forward primer 338F (5′-ACTCCTACGGGAGGCAGCAG-3′) and the reverse primer 806R (5′-GGACTACHVGGGTWTCTAAT-3′) (23). PCR reactions, comprising 25 μl of 2× Premix Taq (Takara Biotechnology, Dalian Co. Ltd., China), 2 μl of each 10 mM primer, and 3 μl of DNA template in a total volume of 50 μl, underwent amplification with the following thermal profile: 94 ℃ for 5 min, followed by 30 cycles of 94 ℃ for 30 s, 52 ℃ for 30 s, 72 ℃ for 30 s, and a final extension at 72 ℃ for 10 min. The resulting PCR products were equimolarly pooled and subjected to paired-end sequencing on an Illumina MiSeq platform with V3 chemistry.

Analysis of the 16S rRNA gene sequences were executed using the bioinformatics software package QIIME2 (version 2021.8) (24). Paired-end reads underwent denoising using the QIIME2 command “qiime dada2 denoise-paired”, which merge paired-end reads, implemented quality filtering, and excluded chimeric and phiX sequences. Taxonomic assignment was performed against the Greengenes (13_8 revision) database using the command “qiime feature-classifier classify-sklearn”. Additionally, an array of alpha- and beta-diversity measures was generated through the commands “qiime phylogeny align-to-tree-mafft-fasttree” and “qiime diversity core-metrics-phylogenetic”. The microbiota community structure between the two groups was analyzed by principal coordinates analysis (PCoA) utilizing permutational multivariate analysis of variance (PERMANOVA) on Bray-Curtis dissimilarity with the R package MicrobiotaProcess (25). The LEfSe software was employed to identify significant differences in microbial taxa, utilizing a linear discriminant analysis (LDA) score >2.0 and *P* value <0.05 (26). Network analysis was conducted using the R package ggClusterNet (27).

### Metabolome sequencing and analysis

The experimental procedure commenced with the thawing of samples stored at −80 °C, followed by a brief 10-s vortex mixing. Subsequently, 50 μl of each sample and 300 μl of an extraction solution (ACN:Methanol = 1:4, V/V) containing internal standards were combined in a 2 ml microcentrifuge tube. After a 3-min vortex and centrifugation at 12,000 rpm for 10 min (4 °C), 200 μl of the supernatant was collected, placed at −20 °C for 30 min, and then subjected to a second centrifugation at 12,000 rpm for 3 min (4 °C). The LC-MS analysis was conducted on a UPLC system utilizing a Waters ACQUITY UPLC BEH C18 column (1.8 µm, 2.1 mm × 100 mm) with a gradient elution.

Data acquisition utilized the information-dependent acquisition (IDA) mode, and the raw LC-MS data in mzML format underwent preprocessing steps, including peak extraction, alignment, and retention time correction through XCMS. Peak area correction employed the “SVR” method, with a threshold for discarding peaks with a detection rate below 50%.

Metabolite identification involved searching the databases. For the differential analysis, VIP > 1 and *P*-value <0.05 were employed. OPLS-DA results, including VIP values, were generated using the R package MetaboAnalystR (28). Prior to OPLS-DA, data underwent log transformation and mean centering, and a permutation test (200 permutations) was conducted to prevent overfitting. Identified metabolites were annotated using the KEGG Compound database and mapped to the KEGG Pathway database. Significantly enriched pathways were determined using hypergeometric test with *P*-value <0.05.

### Proteome sequencing and analysis

Plasma protein extraction was carried out using SDT lysis buffer (4% SDS, 100 mM DTT, 100 mM Tris-HCl pH 8.0). The subsequent steps involved boiling, ultrasonication, and centrifugation to remove cellular debris. The resulting supernatant was quantified using a BCA Protein Assay Kit. For protein digestion (200 μg per sample), the FASP method was employed with trypsin, and the resulting peptides were desalted for LC-MS analysis using C18 StageTip. LC-MS/MS was performed on a Q Exactive Plus mass spectrometer coupled with Easy 1,200 nlC, utilizing a data-dependent top20 method for peptide acquisition.

MaxQuant software (version 1.6.0.16) was utilized for MS data analysis, searching against the UniProtKB database. Parameters included trypsin as the digestion enzyme, 4.5 ppm mass tolerance for precursor ions, and 20 ppm for fragment ions. Carbamidomethylation of cysteines was set as a fixed modification, while acetylation of protein N-terminal and oxidation of methionine were considered variable modifications. Filtering was performed at <1% false discovery rate (FDR) for peptide-spectrum matches and proteins. Label-free quantification employed intensity determination and normalization algorithms.

Protein differences were meticulously examined using the R software, applying a criterion of *P* value <0.05 and an absolute fold change >1.5. Subsequently, GO and KEGG enrichment analyses were carried out for the significantly different proteins. The significance of enriched GO and KEGG terms was established with *P* value <0.05.

Correlation analysis between differentially expressed microbes, metabolites and proteins were performed using Pearson correlation with *P* value <0.01.

## Results

### The gut microbial characteristics within automatic feeding

**Figure 1:**
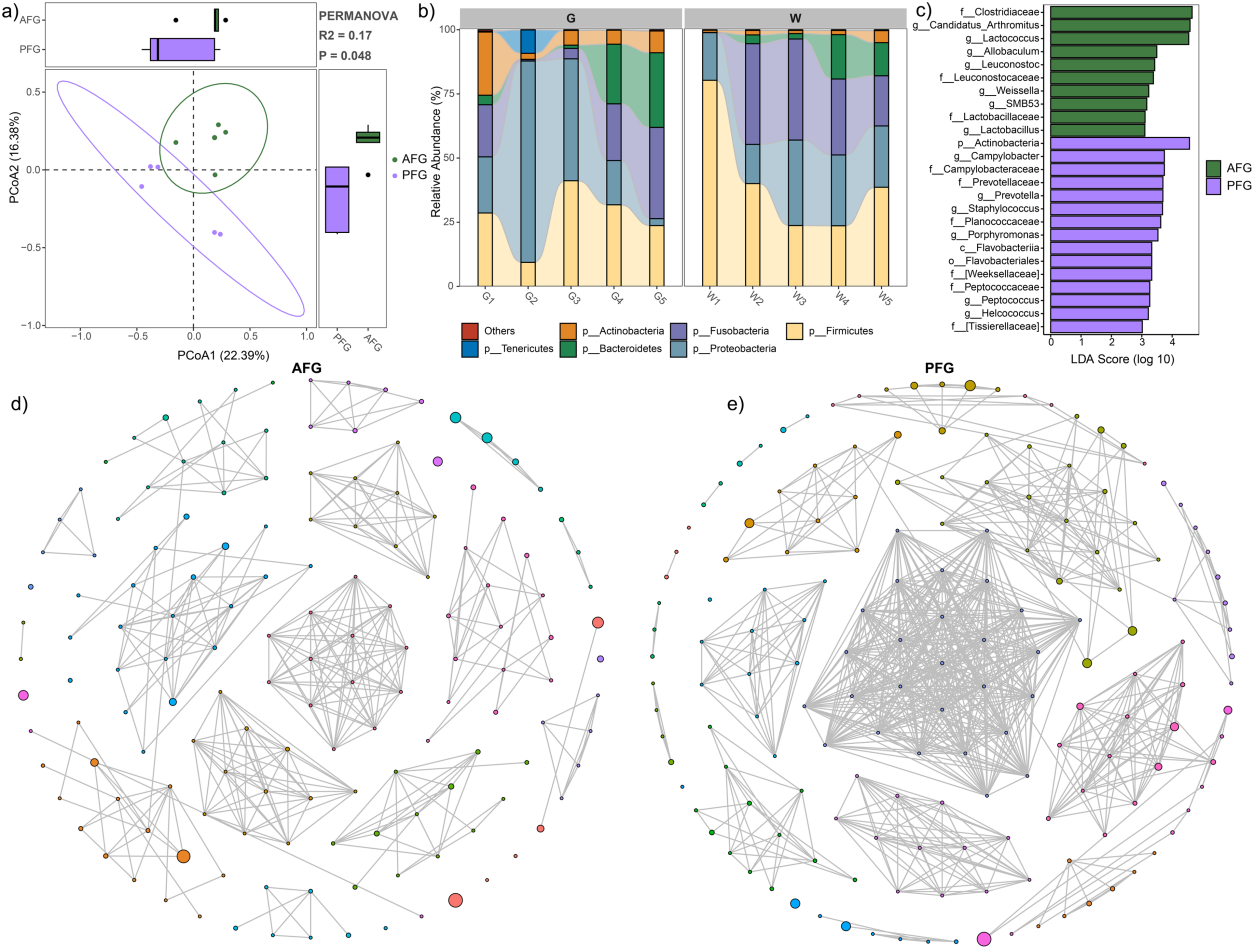
Gut microbial characteristic comparison between the AFG and PFG groups. **(a)** PCoA plot illustrating significant difference between the two groups. **(b)** Stacked bar plot depicting the average relative abundances of abundant phyla in all study subjects. **(c)** LEfSe analysis revealing significant differences in microbial composition between the two groups. **(d)** Gut microbial network of the AFG group. **(e)** Gut microbial network of the PFG group.

The alpha diversity indices, encompassing the observed feature number (1,007.9 ± 355.7 vs. 751.8 ± 420.7, *P* = 0.33), Shannon index (5.32 ± 1.33 vs. 4.77 ± 1.40, *P* = 0.54), and Pielou index (0.53 ± 0.11 vs. 0.50 ± 0.11, *P* = 0.68), utilized to assess microbial richness and evenness within the gut ecosystem, did not reveal significant differences between the AFG and PFG groups. However, a marginal increase in the AFG group was observed. To provide a comprehensive overview of the microbial structure comparison between the two groups, PCoA was employed with PERMANOVA based on the Bray-Curtis dissimilarity. The results indicated noteworthy distinctions in microbial communities (*P* = 0.048, Figure 1a).

The stacked bar plots, depicting the phylum-level composition of microbiota in both groups, were presented in Figure 1b. The prevalent phyla included *Firmicutes*, *Proteobacteria*, *Fusobacteria*, *Bacteroidetes*, *Actinobacteria*, and *Tenericutes* (Figure 1b). Subsequently, LEfSe analysis revealed a higher relative abundance of microbes in the AFG group compared to PFG group. This included the families *Clostridiaceae*, *Leuconostocaceae*, and *Lactobacillaceae*, as well as genera *Candidatus_Arthromitus*, *Lactococcus*, *Allobaculum*, *Leuconostoc*, *Weissella*, *SMB53*, and *Lactobacillus*. Conversely, certain taxa such as phylum *Actinobacteria*, class *Flavobacteriia*, order *Flavobacteriales*, families *Campylobacteraceae*, *Prevotellaceae*, *Planococcaceae*, [*Weeksellaceae*], *Peptococcaceae*, and [*Tissierellaceae*], as well as genera *Campylobacter*, *Prevotella*, *Staphylococcus*, *Porphyromonas*, *Peptococcus*, and *Helcococcus* exhibited higher relative abundance in the PFG group (Figure 1c). Furthermore, to elucidate internal interactions within the gut microbiome, we aimed to identify interactive networks within each group (Figures 1d, e). In comparison to the microbial network in the PFG group, the AFG group exhibited a decrease in the number of nodes (149 vs. 166), number of edges (372 vs. 761), and average degree (5.31 vs. 9.40). These provided insights into the alterations in microbial interactions associated with the feeding interventions, shedding light on potential mechanisms underlying the observed differences in microbial community structure.

### The metabolic characteristics within automatic feeding

**Figure 2:**
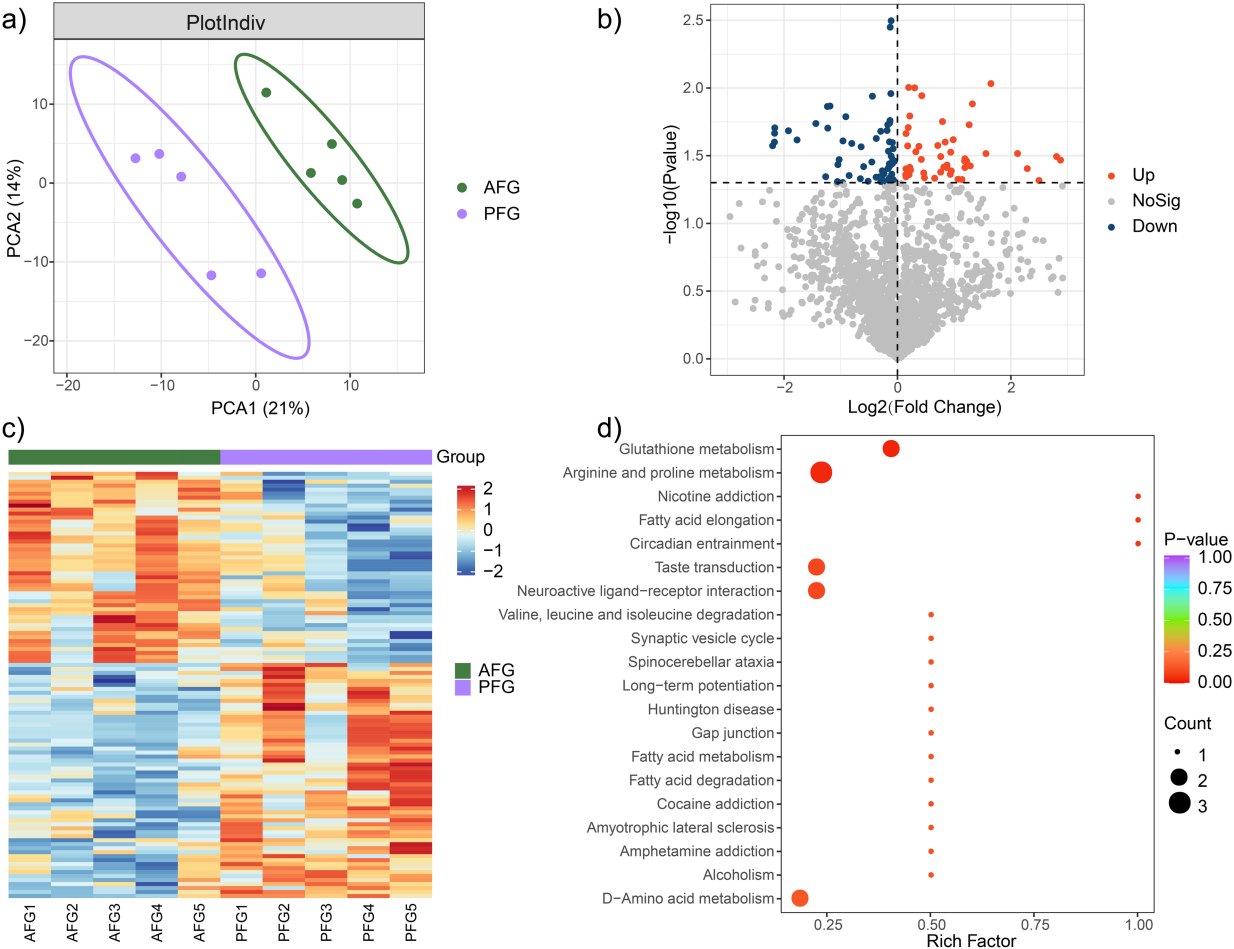
Metabolic characteristic comparison between the AFG and PFG groups. **(a)** The outcomes of the OPLS-DA demonstrated a pronounced and statistically significant separation between the two groups. **(b)** Volcano plot illustrating significantly different metabolites between the two groups. **(c)** Heatmap plot depicting the expression level of significantly different metabolites in every study subject. **(d)** Enrichment functional metabolic pathways involving differently expressed metabolites.

The investigation of the overall metabolic profile between the AFG and PFG groups was carried out utilizing OPLS-DA analysis. The obtained results delineated a significant separation between the two groups with *p* value <0.05 (Figure 2a). Notably, the AFG group manifested 59 down-regulated and 48 up-regulated metabolites in comparison to the PFG group (Figure 2b). The expression levels of these differentially expressed metabolites across individual samples were visually represented through a heatmap in Figure 2c. To gain deeper insights into the pathophysiological implications of the differentially expressed metabolites, a comprehensive functional metabolic pathway enrichment analysis was undertaken. The KEGG pathway enrichment analysis unveiled the involvement of these metabolites in diverse pathways, including but not limited to “Glutathione metabolism”, “Arginine and proline metabolism”, “Nicotine addiction”, “Fatty acid elongation”, “Circadian entrainment”, “Taste transduction”, “Neuroactive ligand-receptor interaction”, “Valine, leucine and isoleucine degradation”, among others (Figure 2d). These results provided a comprehensive view of the potential metabolic alterations associated with the feeding interventions and highlighted the diverse pathways implicated in the observed metabolomic changes.

### The proteomic characteristics within automatic feeding

**Figure 3:**
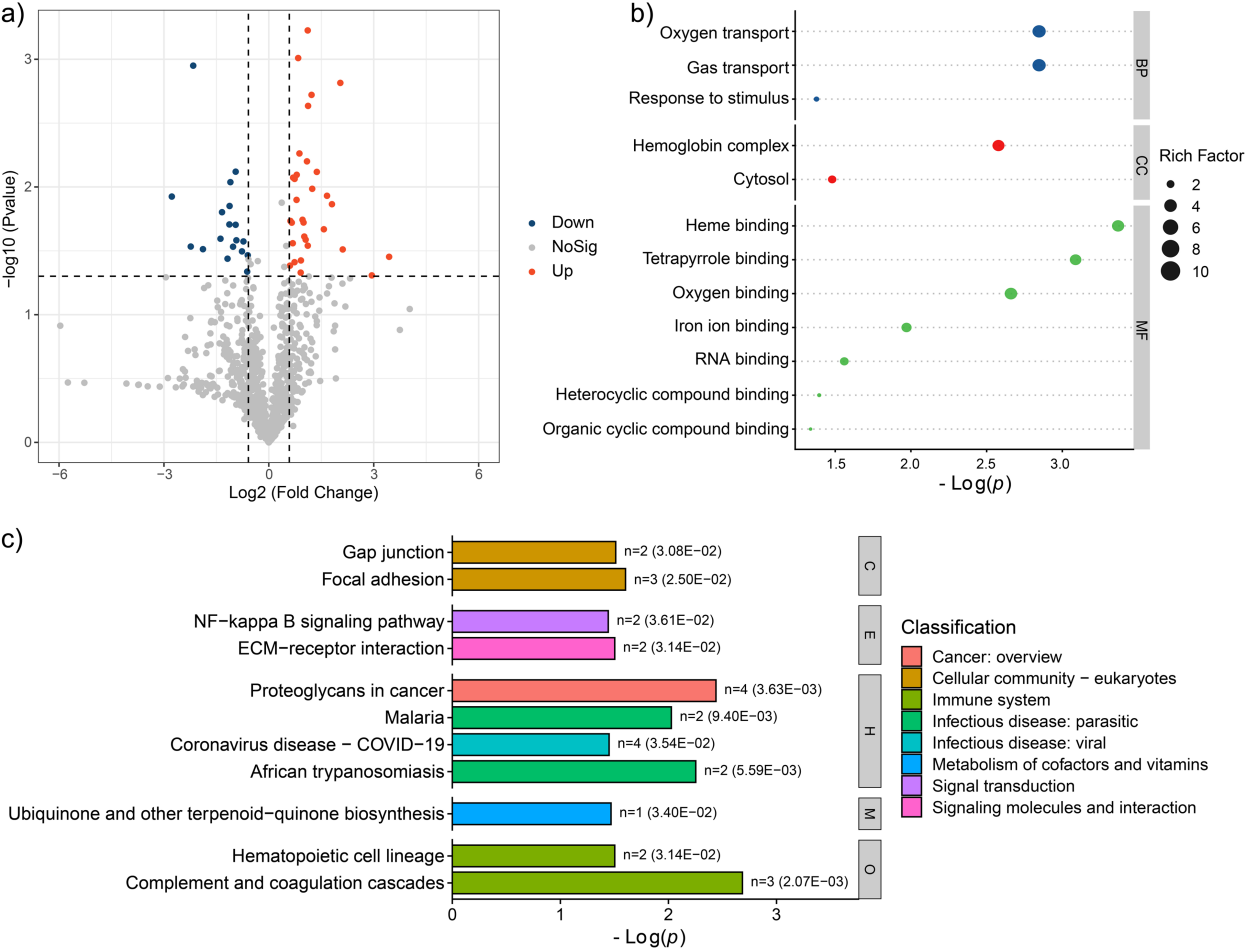
Proteomic characteristic comparison between the AFG and PFG groups. **(a)** Volcano plot showed the significantly different proteins between the two groups. **(b)** GO enrichment terms of the significantly different proteins. **(c)** Enrichment pathways of the significantly different proteins.

Compared to PFG group, the AFG group exhibited 31 up-regulated and 18 down-regulated proteins, as illustrated in Figure 3a. To elucidate the pathophysiological implications of these differentially expressed proteins, a comprehensive functional enrichment analysis was conducted. GO analysis revealed significant enrichment of the differentially expressed proteins in various biological processes, including “Oxygen transport”, “Gas transport”, and “Response to stimulus”. Regarding cellular components, enrichment as observed in “Hemoglobin complex”, and “Cytosol”. In terms of molecular function, the differentially expressed proteins were notably enriched in categories such as “Heme binding”, “Tetrapyrrole binding”, “Oxygen binding”, “Iron ion binding”, “RNA binding”, “Heterocyclic compound binding”, and “Organic cyclic compound binding”, as depicted in Figure 3b. Furthermore, KEGG pathway enrichment analysis unveiled the involvement of the differentially expressed proteins in various pathways, including “Gap junction”, “Focal adhesion”, “NF-kappa B signaling pathway”, “ECM-receptor interaction”, “Proteoglycans in cancer”, “Malaria”, “Coronavirus disease-COVID-19”, “African trypanosomiasis”, “Ubiquinone and other terpenoid-quinone biosynthesis”, “Hematopoietic cell lineage”, and “Complement and coagulation cascades” (Figure 3c). These findings offered a comprehensive insight into the potential proteomic alterations associated with the feeding interventions.

### Muti-omics correlations within automatic feeding

**Figure 4:**
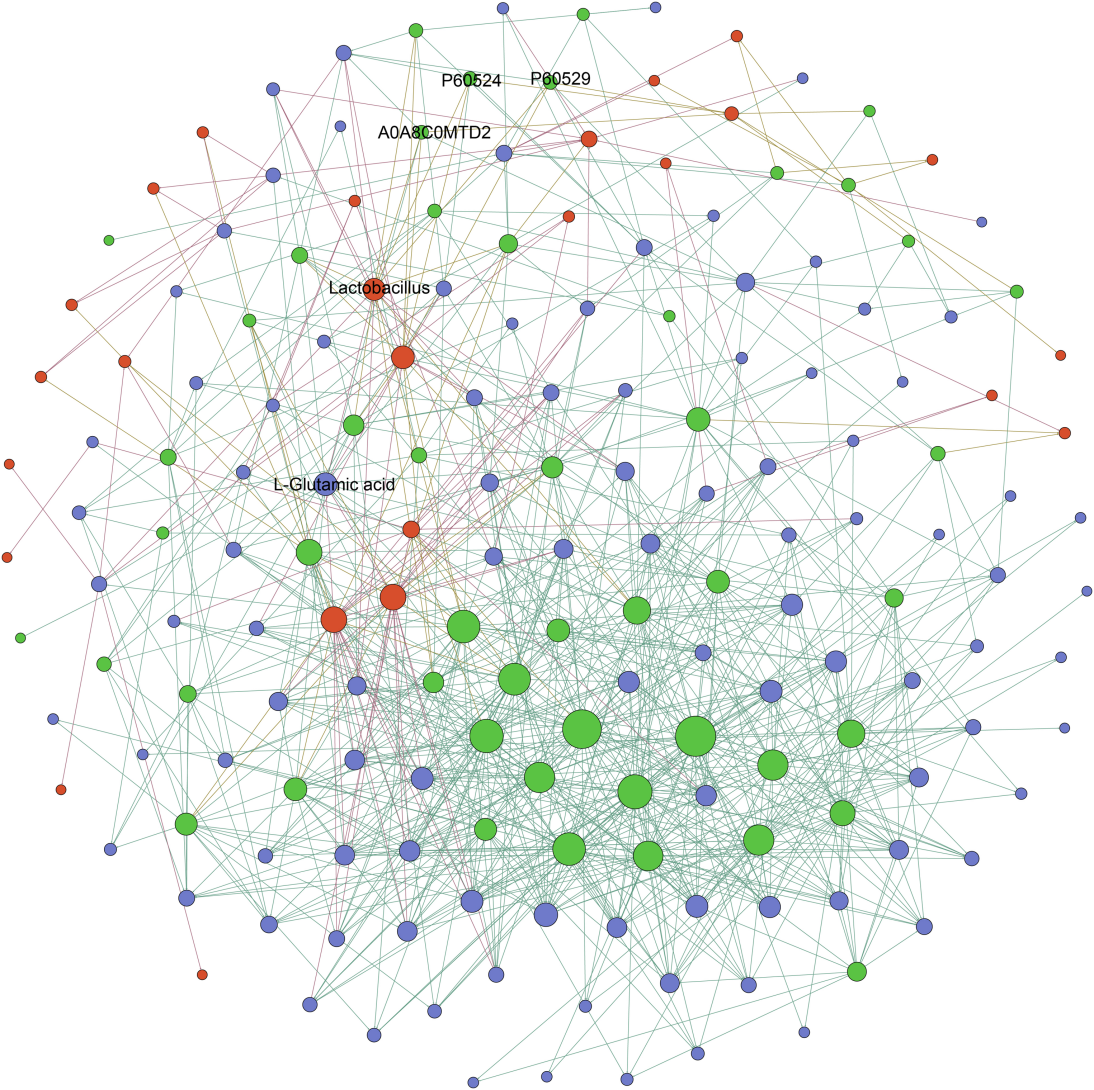
The network diagram illustrating the correlations of differentially expressed microbial, metabolic, and proteomic characteristics between AFG and PFG groups, red nodes represented different gut microbes, green nodes represented differentially expressed proteins, and blue nodes represented differently expressed metabolites.

To investigate the relationships among the differentially expressed microbial, metabolic, and proteomic characteristics between the AFG and PFG groups, the Pearson correlation was calculated. The correlation was identified as *p* value less than 0.01, as depicted in Figure 4. Notably, the proteins enriched in the biological process “oxygen transport”, and molecular function “oxygen binding” included A0A8C0MTD2 (globin family profile domain-containing protein), P60524 (hemoglobin subunit beta), P60529 (hemoglobin subunit alpha). In the correlation network, A0A8C0MTD2 was positively associated with *Lactobacillus* (*R*^2^ = 0.86) and L-glutamic acid (*R*^2^ = 0.77). Moreover, *Lactobacillus* was also positively associated with P60524 (*R*^2^ = 0.91), P60529 (*R*^2^ = 0.81) and L-glutamic acid (*R*^2^ = 0.84).

## Discussion

The advancement and refinement of sucking behavior in preterm infants are pivotal factors influencing their holistic development (1). By detecting the sucking signal within the oral cavity, the automatic feeding delivery device has the capability to administer predetermined volumes of emulsions into the stomach of premature infants, facilitated by a controller regulating the milk supply mechanism. This process is instrumental in fostering the progression of suck behavior. Preliminary data under review, gleaned from animal experimentation, suggests that this device can facilitate gastric emptying. In the context of this investigation, we delved into the impact of the automatic feeding delivery device on beagle dogs, employing a comprehensive analysis encompassing gut microbial composition, metabolic profiles, and proteomic expression data.

A distinctive gut microbial community and network were observed between the AFG and PFG groups, with notable differences in the abundance of various genera. Specifically, the AFG group exhibited higher levels of *Lactococcus*, *Allobaculum*, *Leuconostoc*, *Weissella*, *SMB53*, and *Lactobacillus*, while *Campylobacter*, *Prevotella*, *Staphylococcus*, *Porphyromonas*, *Peptococcus*, and *Helcococcus* were less abundant. It is noteworthy that certain species of *Lactococcus* in the gut have been reported to express *β*-galactosidase, thereby facilitating lactose utilization (29). *Lactococcus lactis* (L. lactis), a primary organism among lactic acid bacteria, is recognized as a safe microorganism capable of regulating the intestinal micro-ecological balance in animals, enhancing host immune function, and modulating brain activity (30, 31). *Lactobacillus* in the gut can similarly reduce lactose concentration through their *β*-galactosidase activity (32). Notably, a study revealed that preterm infants administered with *Lactobacillus reuteri* DSM 17938 exhibited fewer symptoms of feeding intolerance, a common condition in this population (33). Secondary outcomes of the study encompassed the duration of parenteral nutrition, time to achieve full feeding, length of hospital stay, incidence of sepsis, necrotizing enterocolitis, diarrhea, and mortality (33). *Campylobacter*, known for causing infectious disease and diarrheal illness, poses a significant health risk (34). And *Prevotella* has been associated with increased release of inflammatory mediators from immune and stromal cells (35). Collectively, the differential composition of gut microbes underscores the protective role of active feeding facilitated by the automatic feeding delivery device.

Through the metabolome analysis, we identified differentially expressed metabolites that were enriched in pathways associated with amino acid metabolism, including “glutathione metabolism”, “arginine and proline metabolism”, “valine, leucine and isoleucine degradation”, and “D-amino acid metabolism”. Additionally, pathways related to fatty acid metabolism such as “fatty acid elongation”, “fatty acid metabolism”, and “fatty acid degradation” were observed. Furthermore, pathways associated with the nervous system, such as “neuroactive ligand-receptor interaction”, “synaptic vesicle cycle”, “spinocerebellar ataxia”, “nicotine addiction”, “cocaine addiction”, “amphetamine addiction”, and “alcoholism” were enriched. Other pathways, including “circadian entrainment”, and “taste transduction” were also noted. Of particular significance is the role of glutathione in critical physiological processes, with implications for diverse disease pathophysiology (36). Notably, glutathione deficiency is implicated in oxidative stress, a pivotal factor in aging and the pathogenesis of various diseases, including seizure, Alzheimer's disease, and Parkinson's disease (37). Previous study has observed abnormalities in glutathione metabolism in preterm infants with a history of bronchopulmonary dysplasia (38). Arginine, identified as a nutritionally essential amino acid, plays critical roles in spermatogenesis, embryonic survival, fetal and neonatal growth, and the maintenance of vascular tone and hemodynamics (39, 40). Fatty acid elongation, a process predominantly occurring in the liver and other tissues, is crucial for the conversion of fatty acids into longer-chain fatty acids. In preterm infants, this process is incompletely developed, leading to an increased requirement for long-chain polyunsaturated fatty acids (LCPUFAs). LCPUFAs are essential for optimal nutrition, growth, and development, influencing cell membrane structure and function (41, 42). Preterm infants are highly vulnerable to circadian misalignment, mainly due to limited exposure to natural light and the prevalence of artificial light in neonatal intensive care units (43). Enhanced smell and taste perception have been linked to improved milk tolerance, fostering enteral nutrition and facilitating growth in preterm infants (44). Moreover, the autonomic nervous system, especially the parasympathetic division, is relatively underdeveloped in premature newborns compared to the sympathetic division (45, 46). The observed differential expression of plasma metabolites highlights the significant impact of active feeding facilitated by the automatic feeding delivery device on the beagle dogs.

In our proteome analysis, differentially expressed proteins were found to be enriched in GO terms related to “oxygen transport”, “oxygen binding”, “iron ion binding”, “hemoglobin complex”, and “heme binding”. Oxygen supplementation is crucial in neonatal intensive care for preterm infants, aiming to ensure adequate oxygenation for metabolic needs while mitigating the risks of hypoxemia and hyperoxia (47, 48). Heme, an iron-containing molecule, plays a vital role in oxygen transport in the blood and is essential for the development of preterm infants (49). Heme iron serves as a critical nutrient required for infants' growth and development. However, preterm infants are particularly susceptible to iron deficiency anemia due to their limited iron stores at birth, early-onset erythropoiesis, iatrogenic blood loss, and catch-up growth. Notably, enteral iron supplementation has demonstrated significant benefits for preterm and low birth weight infants fed with human milk, including improvements in anemia and linear growth (50). The observed differential expression of plasma proteins collectively underscores the impact of active feeding facilitated by the automatic feeding delivery device on processes related to oxygen transport and heme binding, crucial for the development of preterm infants.

The comprehensive analysis conducted on gut microbial, plasma metabolic, and plasma proteomic data unveiled significant disparities between the AFG and PFG groups concerning the neurodevelopment of preterm infants. Notably, specific molecules, including proteins A0A8C0MTD2, P60524, P60529, genus *Lactobacillus*, and metabolite L-glutamic acid, exhibited correlations with each other through correlation analysis. A0A8C0MTD2, P60524 and P60529 play key role in oxygen transport, and the prolonged periods of hypoxia may precipitate poor growth, cardiopulmonary complications, neurodevelopmental disabilities, or heightened mortality risks in preterm infants (51). L-glutamic acid, a well-established excitatory neurotransmitter with pivotal roles in normal brain function (52), has been implicated in the neurodevelopmental outcomes of preterm infants (53). Emerging research has identified features of gut microbiota as potential mediators for neurodevelopmental and neuropsychiatric disorders in preterm infants (54). Studies have indicated that *Lactobacillus* supplementation can improve the structure of intestinal flora, modulate gene expression, and impact neural development in the brain (54, 55). Zhao et al. demonstrated that an elevation in the relative abundances of *Lactobacillus* could up-regulate the expression of neurotrophic factors and postsynaptic density, thereby inhibiting synaptic ultrastructural damage and alleviating anxiety-like behavior in mice (56). *Lactobacillus*, a type of lactic acid bacteria, has been found to produce L-glutamic acid and gamma-aminobutyric acid (GABA), thereby reducing stress-induced corticosterone and behaviors associated with anxiety and depression (57, 58). The interconnectedness observed among these molecular components highlights their potential roles in shaping neurodevelopment and emphasizes the importance of active feeding facilitated by the automatic feeding deliver device in influencing these critical molecules.

With this study provides valuable insights, certain limitations should be addressed to ensure clinical applicability. The small sample size, limited to beagle dogs, necessitates expansion to enhance statistical robustness. Given the physiological differences between beagles and preterm infants, further research involving clinical trials in preterm populations is essential to validate the device's safety and efficacy. Additionally, examining key phenotypic characteristics of preterm infants, such as body weight, length and neurodevelopmental outcomes, will be better elucidate the device's impact and its interaction with molecular mechanisms in further study. These steps are crucial to transition from preliminary findings to practical, clinically effective applications in neonatal care.

In summary, our investigation has elucidated discernible modifications in gut microbial composition, plasma metabolic profiles, and proteomic expression patterns in beagle dogs subjected to the automatic feeding delivery device. These identified alterations provide significant contributions to the comprehension of the molecular impact exerted by the automatic feeding delivery device. Furthermore, these findings may serve as a foundation for the development of preventative strategies centered on the modulation facilitated by this device.

## Data Availability

The datasets presented in this study can be found in online repositories. The names of the repository/repositories and accession number(s) can be found below: https://db.cngb.org/search/project/CNP0005822.
